# An Open Access future? Report from the eurocancercoms project

**DOI:** 10.3332/ecancer.2011.223

**Published:** 2011-09-12

**Authors:** R Kenney, R Warden

**Affiliations:** European Association for Cancer Research, EACR Secretariat, School of Pharmacy, University of Nottingham, Nottingham NG7 2RD, UK

## Abstract

In March 2011, as part of the background research to the FP7 Eurocancercoms project, the European Association for Cancer Research (EACR) conducted an online survey of its members working in Europe to discover their experiences of and attitudes to the issues surrounding academic publishing and Open Access. This paper presents the results from this survey and compares them to the results from a much larger survey on the same topic from the Study of Open Access Publishing (SOAP). The responses from both surveys show very positive attitudes to the Open Access publishing route; perhaps the most challenging statistic from the EACR survey is that 88% of respondents believe that publicly funded research should be made available to be read and used without access barriers

As a conclusion and invitation to further discussion, this paper also contributes to the debate around subscription and Open Access publishing, supporting the case for accelerating the progress towards Open Access publishing of cancer research articles as a particularly supportive way of assisting all researchers to make unhindered progress with their work.

## Background

In 2010 EACR conducted a survey on professional communication activities across its European membership [1] with particular reference to the use of the internet and barriers to communication. Over half of the survey respondents were working in basic cancer research, a further third in translational research and the remaining respondents in epidemiology or medical oncology. From a range of interesting information and opinions, the survey revealed that the internet is used by 94% of cancer researchers for professional activities every day with the majority accessing PubMed and online journals daily or 2–3 times a week. These simple statistics place access to published research findings online at the centre of support for cancer researchers’ work: a crucial sharing of information which can accelerate progress in the scientific battle with cancer.

While the survey had not focussed on Open Access specifically, comment banks and discussions at consensus meetings following the publication of the survey results highlighted the issue of access to subscription journals, the barrier to essential and urgent information that a ‘paywall’ creates, and the need for free access. Recognising this issue to be an important one EACR completed a second survey picking up on the issue of Open Access publishing in March 2011. This paper publishes the results of that survey, which was again conducted across the European membership of EACR, and cross references responses with selected data from the Study of Open Access Publishing (SOAP) 2011 [2] which was undertaken across all academic disciplines. The SOAP data are freely accessible and can be mined for information by anyone who wishes to. A number of questions were included in the survey that mirrored those used by SOAP, allowing the direct comparison of results. In this article, a comparison has been made between the responses provided by cancer researchers to the EACR survey and the 7,433 respondents to the SOAP survey from the Biological Sciences. (Over 43,000 responses were received across all disciplines to the SOAP survey.)

In 2010, the European Association for Cancer Research (EACR) as a member of the Eurocancercoms FP7 project conducted a survey on professional communication activities across its European membership [1] with particular reference to the use of the internet and barriers to communication. Over half of the survey respondents were working in basic cancer research, a further third in translational research and the remaining respondents in epidemiology or medical oncology. From a range of interesting information and opinions, the survey revealed that the internet is used by 94% of cancer researchers for professional activities every day with the majority accessing PubMed and online journals daily or 2–3 times a week. These simple statistics place access to published research findings online at the centre of support for cancer researchers’ work: a crucial sharing of information which can accelerate progress in the scientific battle with cancer.

While the survey had not focussed on Open Access specifically, comment banks and discussions at consensus meetings following the publication of the survey results highlighted the issue of access to subscription journals, the barrier to essential and urgent information that a ‘paywall’ creates, and the need for free access. A second survey picking up on the issue of Open Access publishing has now been completed. This paper shares the results of that survey, which was again conducted across the European membership of EACR, and cross references responses with selected data from the Study of Open Access Publishing (SOAP) 2011 [2] which was undertaken across all academic disciplines. The SOAP data are freely accessible and can be mined for information by anyone who wishes to use it. A number of questions were included in the survey that mirrored those used by SOAP, allowing the direct comparison of results. In this article, a comparison has been made between the responses provided by cancer researchers and the 7,433 respondents to the SOAP survey from the Biological Sciences. (Over 43,000 responses were received across all disciplines to the SOAP survey.)

As a conclusion and invitation to further discussion, this paper also contributes to the debate around subscription and Open Access publishing, supporting the case for accelerating the progress towards Open Access publishing of cancer research articles as a particularly supportive way of assisting all researchers to make unhindered progress with their work.

## Responses to the survey

The comment banks from the survey can be found in Appendix A.

## Demographics

The professional experience of respondents working in cancer research was well balanced across categories: 31% (18%) had less than 5 years experience, 38% (40%) 5–14 years and 31% (42%) 15 years or more. The figure for the Biological Science respondents to the SOAP survey is given in italics and leans towards greater experience. This is most likely due to the overall profile of EACR membership which includes a high number of students and early career researchers.

## Access to research articles and impact on work

Recognizing the importance of access to published findings highlighted in the earlier report, respondents were asked first how easily they could gain access to peer-reviewed journal articles of interest to their research. A quarter experienced some difficulties or could rarely access the articles they needed. The cancer researchers’ experience was similar to that of the Biological Science respondents to the SOAP survey (29%).

Following this line of inquiry, respondents were asked how often the articles they wished to consult were unavailable because they did not have free access to a particular subscription journal. Over 68% experienced this problem sometimes or often and over 40% could rarely or never compensate by finding the article in an Open Access repository. As a result of this situation, almost 59% of respondents indicated that a lack of access sometimes or often slowed down their work.

The comment banks also revealed that research is being hindered by a lack of access to the articles that are required and that valuable time is spent on attempts, sometimes futile, to find what is needed. Comments show that some solutions could be found informally but required resourcefulness, persistence and placed great reliance on informal international networks of friends and colleagues.

## Experience of and attitudes to Open Access publishing

Respondents were then asked about where they were published and their experience of and attitudes to Open Access publishing.

When respondents were asked how many peer-reviewed research articles they had published in the last five years (Open Access and not Open Access), 41% (39% SOAP) had had 1–5 articles published but there were also 33% (42%) with 6–20 articles published and 13% (13%) had published more than 20 articles; 12% (5%) of respondents had not published in the last five years.

Fifty-eight per cent (65%) had published 1–5 research articles in Open Access during the same period, and 15% (11%) had published 6 or more. The proportion who had not published in Open Access during the same period amounted to 27% (19%).

The distinct similarities between the EACR and the SOAP survey data are more significant than the relatively minor variations, variations that are likely to be due to the relatively more experienced profile of the SOAP respondents which were alluded to earlier. With 73% of EACR respondents already publishing in Open Access journals, there is clearly growing acceptance of and engagement with this route to publication.

## Choosing where to publish and why

When respondents were asked which factors were important to them when selecting which journal to submit to for publication, a combination of ‘Journal Impact Factor’ and the ‘Prestige/Perceived Quality of the Journal’ was seen as the most important. Over 95% (94% and 90%) regarded these issues as important and more than 50% indicated (48% and 41%) that they were ‘extremely’ important.

These factors were closely followed by the importance of the journal to academic promotion, tenure or assessment with more than 85% (73%) highlighting this factor as important. At 85% (90%), this rating was matched by the relevance of the journal for the researcher’s community. Also important to respondents with scores of over 80% were the speed of publication of the journal, the likelihood of acceptance and a positive experience with the publisher/editor of the journal.

Over 30% regarded the policy fit of the journal with the researcher’s organization, journal copyright policy and personal recommendation of the journal as important factors, with ‘irrelevant’ ratings never exceeding 25%.

The Open Access nature of the journal was seen as important by over 40% when choosing a journal to publish in, and less than 10% regarded this as irrelevant.

These are particularly interesting statistics as the three factors cited as most important in choosing a journal are related to impact, high esteem and quality: factors that reflect well on the author and support job security and career progression. Unsurprisingly perhaps, only a handful of respondents see any of these factors as irrelevant. The choices are pragmatic and well embedded in the professional culture.

Set against this, the score for Open Access offers encouragement for those who hope to see this form of scholarly publishing continuing to grow quickly in the future. Very few cancer researchers see the choice of Open Access as irrelevant and approaching half see it as important. This reflects the earlier concerns about the difficulty of access to articles as a reader when you need the information to advance your work and suggests that if Open Access can address any remaining concerns over esteem and quality, authors will progressively move across to this model.

## Securing access

Money to meet publication fees and the opportunities for publication in Open Access journals come from a variety of sources; 33% of respondents were able to publish without being charged a fee, 23% had money for publication included in their research funding and a further 24% had their fees paid by their institution, and 4% of respondents paid the fees themselves. SOAP did not enquire about Open Access publication without paying a fee so direct comparison of data is not possible here. However, it was also only a small percentage in the SOAP data that met their own costs (6%).

The EACR survey went on to explore how many researchers were mandated by their funding body to publish in an Open Access repository after a certain time had elapsed following publication in a subscription journal. Only 13% indicated that they were required to follow this route, with the others divided between those who were not required to follow this route and those replying ‘Don’t know’. Of the small number required to comply, a third found it difficult or very difficult to do so.

The survey also explored how long an embargo was considered reasonable before an article published in a subscription journal could be placed in an Open Access repository; 71% of the respondents were closely divided between suggesting the reasonableness of 3- and 6-month embargos with 17% of respondents believing that it should be the publisher’s decision when to release.

## Attitudes to the principle of Open Access

The most challenging statistic from the EACR survey is that 88% of respondents believe that publicly funded research should be made available to be read and used without access barriers: a figure just a little lower than the (90%) score recorded by the ‘Biological Sciences sample’ in the larger SOAP survey. Such a high score is a direct challenge to those involved in subscription publishing, as are the responses that indicate attitudes to Open Access are very positive. Respondents feel that articles that are available by Open Access are likely to be read and cited more often than those not Open Access 73% (67%), with only 10% (11%) disagreeing; 77% (74%) believe researchers should retain the rights to their published work and allow it to be used by others.

There are a number of key areas of concern in respect of Open Access publishing, and paramount among these is the perception that paying publication fees will mean less money available for research 66% (53%) and that this could penalize research intensive institutions with a large publication output by making them pay high costs for publication 40% (28%). However, only 19% (11%) and 22% (14%), respectively, believe that Open Access publishing undermines the system of peer review or leads to the publication of poor quality research.

## An Open Access future?

There is only one real reason why most publishers have not fully embraced Open Access publishing as a business model and moved from the subscription model: Open Access will not and cannot generate the level of income and profit that is presently produced by the current business model.

The traditional subscription model is simply too attractive to publishers but completely out of balance for the funders of research, the authors of research articles and the subscribers:

- Cancer research is funded by the public through taxation or charitable donations. The output is then published with no requirement on the publisher to compensate the author or funder of the research.
- A peer review process is essential, and the cooperation of senior researchers in various capacities is essential to the process.
- Contracts between the author and the publisher are drawn up that protect the publisher’s interests by securing the article behind a ‘paywall’.
- Public money comes into play again as journal subscriptions are purchased by librarians and resource managers. This provides selective access for most but by no means all of the researchers active in cancer research.
- Subscriptions provide online access not only to newly published papers but to the archive as well, but if the archived articles have not been placed in an Open Access repository following a period of embargo or for any other reason, access to the archive will be lost by the subscriber if the subscription is cancelled.

No reasonable person would deny that the investment of the major publishers in the IT infrastructure for their journals is impressive and that a return is required for such an investment, but that return does not have to be secured through the restrictive practices of ‘paywalls’ and copyright licenses.

Open Access journals may offer free publication and access if provided with a funding stream from a foundation or other interested organization but many will only be able to function if a publication fee is charged. However, there should not be any difficulty with a ‘pay to publish’ model if,

- The research funder includes money to meet publication fees if they are required.
- The publisher accepts that the income from journal publication will fall but that there is ultimately no alternative as the landscape and expectations are rapidly changing in the digital age—not as dramatically yet as in access to recorded music and news publishing, for example, but the changes will not go into reverse. Publication fees will provide income and perhaps we can trust, at least initially, that free market competition will keep downward pressure on publication fees.
- Publication costs are defrayed in some manner by website advertisements or by using a ‘community’ as a basis for market research as is the case with the website Doctors.net.uk

The ‘Green route’ to Open Access offers the opportunity to see more articles reach those who need to read them but only after a period of embargo imposed by the publisher of a subscription journal. Not all publishers allow this route to be taken and where they do, legal complexities around copyright and licensing abound, with the definition of which version of a paper may be released post-publication being just one.

Authors themselves can do without the additional burden of managing the journey of their article into the Open Access domain after the publisher’s embargo period—and many apparently do, as underlined by Robert Kiley [3] who indicated that only 43% of mandated researchers presently observe the requirements that have been set by the Wellcome Trust.

The ‘Hybrid’ model also has a superficial allure where established subscription journals accept articles on an Open Access basis with a publication fee for inclusion. However, it is certain that an unbalanced business model will obtain where librarians, and others paying the subscription charge of a journal, will be buying access to some journal content to which access has already been purchased using public money. The Wellcome Trust is one body that has expressed its concerns about Open Access fees being paid twice [4].

The publisher may of course on grounds of fairness reduce the price of the subscription proportionately, as has been done by OUP for its hybrid journals [5] and for the EMBO Journal and EMBO reports published by NPG on behalf of EMBO [6], but there has been no great move in this direction. As Stephen Pinfield points out, ‘As publishers’ income has increased from OA fees in the hybrid model, there has been little or no let up in journal subscription inflation, and only a small minority of publishers have yet committed to adjusting their subscription prices as they receive increasing levels of income from OA options’ [7].

## Is an Open Access future inevitable?

Publishing of cancer research articles is a valuable service provided by publishers. However, publishers are driven by the profit motive and must necessarily satisfy their shareholders. Their business is entirely dependent on public money, and that from the charitable giving, that funds the research and then pays for the published results.

In ‘Will open access compete away monopoly profits in journal publishing?’, Bergstrom and Bergstrom present a concise and cogent view of the ethical and economic argument for Open Access publishing stating in their conclusion, ‘A powerful technological reality looms over this entire discussion. With electronic access, the marginal cost of allowing an extra person to read a scholarly work approaches zero. When publishers—even non-profit operations interested in maximizing circulation—rely on subscriptions to generate revenue, distribution is inefficient because potential readers are excluded though it would cost nothing to allow them access. Open access publishing is one way of realizing the enormous potential gains that the internet offers’ [8].

In ‘The Access Principle – The Case for Open Access to Research and Scholarship’, John Willinsky states that ‘commitment to scholarly work carries with it a responsibility to circulate that work as widely as possible’. He sees that in the digital age, that responsibility includes exploring new publishing technologies and economic models to improve access to scholarly work. He argues that Open Access benefits all, from the established and well-supported researcher to those struggling hard to find resources [9].

Change is inevitable as funders challenge the extraordinarily restrictive and profitable business models and choose different directions. A positive recent example is the approach to be taken by three important partners.

‘The Wellcome Trust, the Max Planck Society and the Howard Hughes Medical Institute are set to launch an open access research journal that will attempt to compete directly for submissions with *Cell*, *Nature* and *Science*. They will publish the first issue of the as-yet unnamed online only publication for biomedical and life sciences research in summer 2012. Authors will not be charged fees and anonymous reviewer comments will be published …. It is unusual for research funders to get involved in journal publishing. The group says that the move is an attempt to resolve scientist frustration with the publishing models used by the top journals’ [10].

There is a moral imperative for Open Access, the internet provides the platform for its realization and funders, and other interested bodies can provide the means to accelerate change.

The responses from the cancer researchers in EACR, particularly when married to the complementary data in the SOAP survey, give significant support to a new model that will allow researchers to access the latest published findings in their community as soon as they are available. The strongly supported view that publicly funded research articles should not be placed behind a ‘paywall’ should also help maintain the momentum for change. One doubts that members of the general public, funding cancer research through taxation and charitable giving and hoping for advances to combat the disease, would be any more sympathetic to the restrictive practice of subscription publishing.

## Data

The full survey data can be found on the EACR website at: http://eacr.org/about/eurocancercoms.php

## Figures and Tables

**Figure 1:**
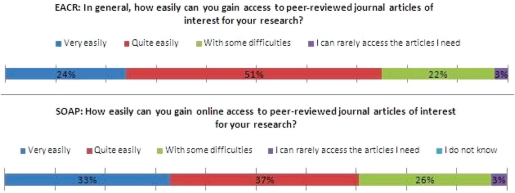
Access to articles of interest for research.

**Figure 2:**
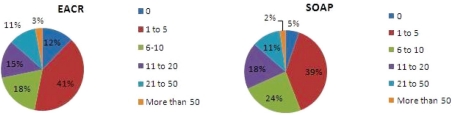
How many peer-reviewed research articles have you published in the last five years?

**Figure 3:**
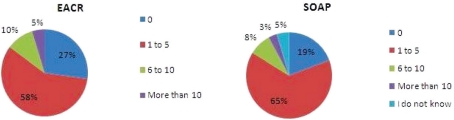
Approximately how many of the research articles you have published in the last five years were published in an open access journal?

**Figure 4:**
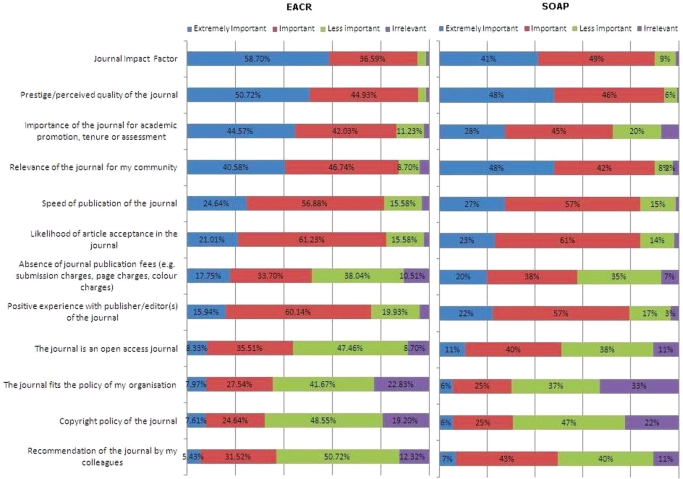
What factors are important to you when selecting a journal to publish in?

**Figure 5:**
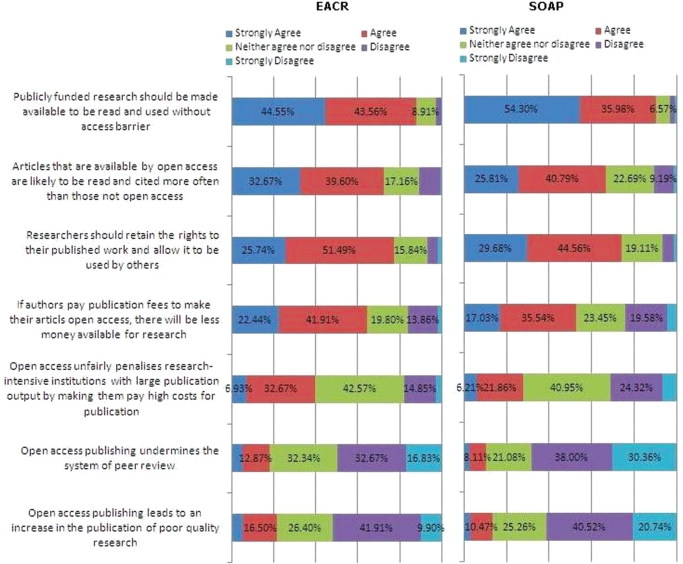
Attitudes to the principle of Open Access.

## Appendix A Survey comment banks

**Table:** EACR Open Access Survey March 2011: Comment Banks

(In general, how easily can you gain access to peer reviewed journal articles of interest for your research?)	
(If you answered ‘With some difficulties’ or ‘I can rarely access the articles I need’, please answer the below)	
What is the impact of these difficulties on your work?	
1	We have no access to several journals
2	Sometimes I can’t access the articles that I need
3	It takes more time to find the ways how to get the article I need
4	Important papers that would help with my research cannot be accessed which makes it difficult to troubleshoot problems
5	Pretty high
6	It requires more time to get the article
7	I do not cite an article that I don’t access
8	Minimal, although it can be annoying
9	Can’t have free access to some of needed papers
10	I am a regulatory & scientific writer. When working with clients, I need quick access to many published articles in order to build the document I am working on. Since I am an independent, non-academic worker, I do not have easy access to all the articles I need and this certainly impedes my work.
11	High
12	Very much impact
13	It slows down the initial pre-project research, sometimes a lot of time is lost on trying to access just one paper
14	Slower collecting information
15	Sometimes the article I can’t access is just the one I seek
16	Slows down work
17	Sometimes papers with potential relevance are not taken into account
18	Insufficient data for experiment planning
19	Our institute has subscriptions for lot of journals. However these subscriptions are not timely (generally with 1 year embargo)
20	I spend more time looking for articles.
21	I miss scientific information
22	The impact is high, it debilitates my ability to plan my research and to interpret my results in the context of other results in the field
23	Quite heavy
24	Often I cannot get access to the journal I needed to read
25	Some vital data from such articles often impede me from understanding the most of the topic
26	It delays my thinking
27	It takes me more time to find the similar or relevant issues
28	Do not have a quick and free access to the journals you might need
29	Delay in keeping informed on latest advances
30	I need much more time to find necessary articles so that makes me to work very slowly
31	I have to wait longer to read desired article, but not longer than a week
32	It holds things up and you are being discouraged of following some lines of interest
33	At my work place there is a limited access to online articles, and the speed of the connection also poor. However I have a licence to a great online university library, this is related to one certain computer, which is at home
34	It has a substantial impact on my work
35	Only access through university
36	I am using university web server and it has limited allowance to access articles from Pubmed
37	My university does not provide access to specialized journals
38	It is too expensive for our administration to buy access as we only are one research group at the foundation
39	Very high, lost lot of time
40	Very high because sometimes it is really important to get the article you’re looking for to continue your work!
41	Lack of information
42	I have to order them through our library
43	Not having free access to articles means that I use to much time on saving articles in paper copies, as I can have most as hard copies but not as pdfs
44	Loss of time and information
45	Decreases competitiveness
46	Relatively often I come upon an article in the journal to which my institution does not have access
47	I can almost never access the most recent articles (with less than one year)
48	Pretty big
49	It holds in delay
50	Limiting and time consuming
51	Sometimes I don’t have access to the journals and then I have to ask a colleague from the university to send me the pdf. Most of the times I do not read the article unless it is very important for my work
52	Longer period of time for synthesis and analysis of data
53	Makes it difficult to gather necessary background information for my work
54	Delays the rapid acquisition of up to date knowledge
55	Slows down my work
56	Slows things down, miss some papers
57	Time consuming
58	I can’t learn everything about my works. There are some problems or questions and I can’t find the answers sometimes
59	Some delay
60	Loss of time (trying to access the same/similar information from other sources) Need to do experiments, to repeat the procedures to get the inaccessible results interestingly, make contacts, to be able access the articles by the help of other researchers in other institutions
61	It makes complicated to access to important new results, but I get them anyway
62	Financial difficulties
63	No subscription to Oncogene and other journal of interest
64	I don’t have full information on my subject because the most helpful articles usually require payment
65	Access to some articles involves re-routing through different publishing sites. Some of which are not accessible via our Library or via PubMed due to restrictions and the need to pay for them before accessing them
66	Great impact, as we need to get updated continually
67	Delay of time
68	To some extent, they slow it down
69	I don’t get all articles I want to read
70	We have not open access to some journals
71	Has a major impact
72	Many Journals require different fees for the reading of the publications
73	It has impact on my job for timely following up the recent research activities
74	Time consuming, costs or less informed than I wish. I used to be a researcher and then my access was good. As a person now working with funding, it is not that critical
75	A big impact because we cannot correlate our research with others
How are you able to find a way round this problem?	
1	We ask the authors about copies
2	Ask colleagues with access
3	I try to contact colleagues who have access to large databases and can send me the articles
4	Can ask other institutes if they have access to a particular journal
5	Requesting articles directly from corresponding author
6	I pay to get access
7	Asking other colleagues who have access
8	I can access most of them at the university, if I can’t and if I need the article, I purchase it
9	Ask for a PDF
10	I ignore the research - it will impact on the author’s citation rate
11	Pay for it
12	I go to a medical library nearby and Xerox or print out the articles I need. Of course, these are nonelectronic copies which do not allow me to cut and paste what I need, so I have to copy/paraphrase sections by hand-quite in the old fashion!
13	Ask friends who have access to the articles
14	Requested article from authors by email
15	Contact co-workers from other countries to download the paper and send it to us
16	It depends - mostly with the help of other colleagues
17	e-mailing corresponding authors - they usually answer and send back the article or at least proof of the article
18	Library loans
19	Go the slow way and ask for pdfs or get paper copies
20	Ask colleagues from foreign institutes to send the article
21	Generally by friends that are Msc or PhD students in various institutes
22	I usually ask one of the authors for a copy of the article
23	I ask for papers from other friends
24	If the paper is really pertinent I ask colleagues working at other institutions that have institutional access to that particular paper to download it for me and send it to me but I feel uncomfortable doing it for all the papers that would like to read
25	I ask friends to help me
26	it is time intensive and often I decide not to bother to read the full article
27	I usually write to the corresponding authors, requesting reprints
28	I request reprints from authors or ask my friends over the worlds to send me PDF copy
29	I am asking the authors to email me their article(s)
30	Not so often
31	Sometimes you need to buy these data or journals by yourself
32	Ask help from other Institutions
33	I try to contact my professors or friends who maybe have these articles I need but this is not a solution
34	I ask my boss’s secretary to download article for me
35	If you are seriously interested in an article, you have to ask colleagues or the authors
36	I write e-mails to the authors to get the papers I need
37	Open access journals, however, there will be the problems, when I want to submit the paper
38	Students may access with a certain amount of membership fee just for this issue
39	Directly contact the authors
40	I need to contact friends to get access
41	Asking for help from my colleagues
42	Asking someone else...but without any success!
43	Asking the author for a copy or asking a colleague that works in another institution (and country) and has access to the article for help
44	My office is full of paper!
45	That’s the question; seeking by deviating ways to get electronically access to the journals’ informations
46	Ask friends for papers
47	I write to authors, and if that does not help, I ask my friends who work abroad to send me the pdf copy
48	Sometimes I send direct emails to the authors, asking if they can send me the papers
49	Requesting directly from corresponding author
50	I send a message to author of the article and wait. Sometimes I have the article
51	Continue searching other article I have access to under the same subject or through people who have access
52	I ask a colleague who works in the university and that has access to more journals
53	By sending email to the lead author of the paper with a plea to send me article
54	Sometimes by writing to the authors and asking for a copy of their paper, but it doesn’t always work
55	Don’t read more than the abstract or I hassle to get a copy
56	Persistence
57	Relying upon the abstract only
58	Spend more time reviewing the literature do actual experiments write e-mails and wait for response
59	When I can’t find a paper I want, then I try to find someone likely to have access to the journal - often this involves an email
60	Sometimes through my librarian
61	Through medical representatives of pharma industry
62	Trying to use access of other organisations
63	Support from Russian Fund of Basic Researchers and other funds
64	Open access for more articles
65	Find alternative papers close to the subject by the same author. Ignore the paper. E-mail a request direct from the author
66	Sometimes I ask friends who have access to certain journals to send me the articles
67	I have to wait or I mail to the authors directly
68	Through friends
69	I ask authors to send me a copy of their articles
70	We ask the authors to send copies
71	Referring to other researchers, seeking help from other research centers
72	1.) I ask my previous students, who are working in American or EU research institutions to e-mail the publications to my address. 2.) I used to contact the first authors of the publications, and ask them to mail to me the sample copies or proofs
73	Some I can access in my university, for some others I buy
74	I can get articles from colleagues
(Does your funding agency require published articles to appear in an open access repository after a certain time has elapsed after publication (e.g. 6 months)?)	
(If you answered yes, please indicate how easy you find it to comply with this requirement)	
If you answered that it is ‘Difficult’ or ‘Very difficult’ for you to comply, please indicate why you find it difficult (e.g. too busy, difficult to do, etc)	
1	Conflicts with journal policies unless substantial fees are paid, no institutional open access repository for manuscripts
2	Additional bureaucracy, additional fees, more hassle
3	No extra money and some journals charge a great deal of money to offer open access
4	Restricts journal choice, lack of funds after grant finishes, no funds provided by grant
5	There are many colleagues, not only from pure countries, who ask for copies of my papers
6	Difficult to do. Unclear what journal policy is regarding articles appearing in an open access repository
7	Costs
8	Extra costs involved
9	Sometimes the proposed experiments does not finish on deadline of grant and the funding organization ask for regular report
10	Because the funds are limited, and there are many competitors for the money
11	Difficult to do
If you have any further comments to add, please include them below	
1	There is confusion as to what Open Access means. I noticed there are two definitions used in my institution: 1. open access: immediately accessible to the public; copyrights with authors; high publication fees; and 2. embargo for subscribers, publicly available after 3–6 months, lower publication fees. Both are great. I only have a problem with journals that do not make their articles available after 6 months.
2	There are +’s and −’s to this question. Perhaps open-access could become standard practice at a certain time after publishing (i.e. after 1 year or 2 years...?)
3	Cost is prohibitive, as are colour page charges etc. We have to look carefully at the journals’ websites to find this out before deciding where to submit. There just is no money for this sort of thing
4	I think it is important to consider that there are really two (at least) types of Open Access journals, the worst type is just money making (some established paper journal publishers are not much better, in fact!). An important point is that some important and scientifically sound research just doesn’t pass the ‘novel, but not novel enough’ and ‘this can’t be true until found by one of the big names in the field’ hurdles. Open Access can partly overcome these and that may be its most positive impact
5	Science shouldn’t be a luxury
6	Research should rely on public founding only, results should be peer reviewed, and made available to the community for free
7	Unfortunately, scientific publishing is big business
8	Publishing should not be a business
9	I find it difficult to agree with the concept that I have to pay to publish my own research. I believe that either institutions have to have a policy to cover publication costs, as they don’t have to pay subscription costs, or publishers should retain rights and cover their costs through subscription. Open access fees can be restrictive for academics that have high quality results but no funds to cover the often expensive publication costs
10	Thank you
11	I support open access publishing but the costs are very high and often funding is not provided for that -- journals should make less of a profit!!
12	I am very much for open access. The huge subscription fees should then disappear. This money can go to the scientists to cover the publication fees. I think impact factors are inhibiting science rather then stimulating it and they should disappear. I am very much for the PLOSONE formula!!!! No more submitting to multiple journals - that will save time
13	I think that restricting papers/research access to developing countries by demanding an upfront payment is morally wrong. It makes some work elitist
14	None
15	I would expect that increasing use of open access would lead to a shift of funds from library budgets to researchers’ budgets. Whether there would be a net increase or decrease of money to publishers, I cannot say
16	I am now 70 years old. 48 years have been spent in virological research. There is an advantage offered by researchers publishing in free access journals or who are rich enough to pay fees for publications. I understand, that the publishers have to make profit, but the present system is significantly inhibiting the fair development of research and researchers
